# Scarf pin inhalation: clinical characteristics and surgical treatment

**DOI:** 10.1186/s13019-015-0268-z

**Published:** 2015-04-26

**Authors:** Hicham Fenane, Mohammed Bouchikh, Khalid Bouti, Mehdi EL Maidi, Fahd Ouchen, Tchely-Oaly Mbola, Lamboni Damessane, Abdellah Achir, Abdellatif Benosman

**Affiliations:** 1 Department of Thoracic Surgery, Ibn Sina University Hospital, BP 353, Rabat Principale, 10001 Morocco; 2 Unit of pedagogy and research in Thoracic Surgery, Faculty of Medicine and Pharmacy, University Mohamed V, Rabat, Morocco; 3 Respiratory Department, Regional Hospital Center of Tetuan, Tetuan, Morocco

**Keywords:** Hairpin, Foreign body, Inhalation, Bronchoscopy, Thoracotomy, Epingle, Corps étranger, Inhalation, Bronchoscopie, Thoracotomie

## Abstract

**Objective:**

Scarf pin inhalation is becoming a frequent accident among young Moroccan woman who wears islamic veil. The aim of the study is to highlight indications, principles and challenges of surgical removal of that particular foreign body.

**Methods:**

Twenty-eight patients were hospitalized in Thoracic Surgery department of Ibn Sina Hospital at Rabat between January 2008 and June 2013 for surgical removal of a pin scarf after unsuccessful endoscopy.

**Results:**

Mean age was 20 years. Inhalation was accidental in all cases. Average interval between inhalation and surgery was 10 days. Penetration syndrome was found in 82% of patients. Pin was located at the left tracheo-bronchial tree in 53.5% of cases and at the right one in 46.4%. All were operated by thoracotomy. Surgery was conservative in all cases, and postoperative course was uneventful.

**Conclusion:**

In case of failure endoscopic treatment, surgery remains the only alternative. It must be as conservative as possible. Short interval between inhalation accident and surgery prevents parenchymal resection.

## French abstract

**Introduction:**

L’inhalation d’épingle à foulard est un accident de plus en plus fréquent chez la jeune femme marocaine qui porte le voile islamique. Le but de ce travail est de mettre le point sur indications, les principes et les difficultés de la chirurgie de ce corps étranger particulier.

**Méthodes:**

Vingt-huit patientes étaient hospitalisées au service de Chirurgie Thoracique de l’hôpital Ibn Sina de Rabat entre janvier 2008 et juin 2013 pour extraction chirurgicale d’une épingle à foulard après échec de l’endoscopie.

**Résultats:**

La moyenne d’âge était de 20ans. L’inhalation était accidentelle dans tous les cas. Le délai moyen entre l’inhalation et la chirurgie était de 10 jours. Le syndrome de pénétration était retrouvé chez 82% des patientes. L’épingle était localisée au niveau de l’arbre trachéo-bronchique gauche dans 53,5% des cas et à droite dans 46,4% des cas. La voie d’abord était une thoracotomie et aucune résection parenchymateuse n'était nécessaire.

**Conclusion:**

La chirurgie ne doit être une alternative au traitement endoscopique qu’en cas d’échec de ce dernier. Elle se doit d’être conservatrice autant que possible. Le court délai entre l’accident d’inhalation et la chirurgie permet d’éviter les résections parenchymateuses.

## Background

Foreign body (FB) inhalation is a serious problem that usually happens to children [1,2]. The type of FB depends on regions, eating habits and even clothing habits. Scarf pin is a particularly common FB in Islamic countries where women wear veil [3,4].

We present a series of 28 cases of scarf pin, operated in Thoracic Surgery Department of Ibn Sina hospital in Rabat. We would like to highlight indications, principles and challenges of surgical removal of this FB.

## Methods

Between January 2008 and June 2013, 28 patients were operated in our department for surgical removal of a scarf pin inhaled by accident.

Average age was 20 years. No favoring factor (swallowing disorder, neurologic, neuromuscular or metabolic disorder) was found. All patients received a chest radiograph to localize the radio-opaque pins. Radiography was repeated right before surgery to verify possible migration of the pin.

Patients requested surgical treatment after repeated, unsuccessful flexible bronchoscopy removal, on an average 2.5 attempts per patient. Seven patients underwent rigid bronchoscopy as well. This failure was due to very distal migration of pins and/or their embedding in the bronchus wall. The patients received antibiotics, and in 10 cases an additional short-term steroid treatment was included.

The study was approved by the ethics committee of the unit of pedagogy and research in Thoracic surgery at the Faculty of Medicine and Pharmacy of Rabat, Morocco.

## Results

The foreign body was a 2–3 cm long metallic scarf pin, pointed on one end and with a spherical plastic cover on the other. Inhalation happened accidentally while arranging the scarf with both hands and holding the pin by its plastic end between teeth. Our patients reported that they were speaking, coughing, laughing or taking a deep breath when the accident happened.

Average delay between inhalation and surgery was 10 days, the shortest period being 3 days and the longest 21 days. Penetration syndrome was found in 23 patients, meaning 82% of cases. Slight hemoptysis was reported in 6 patients.

**Figure 1:**
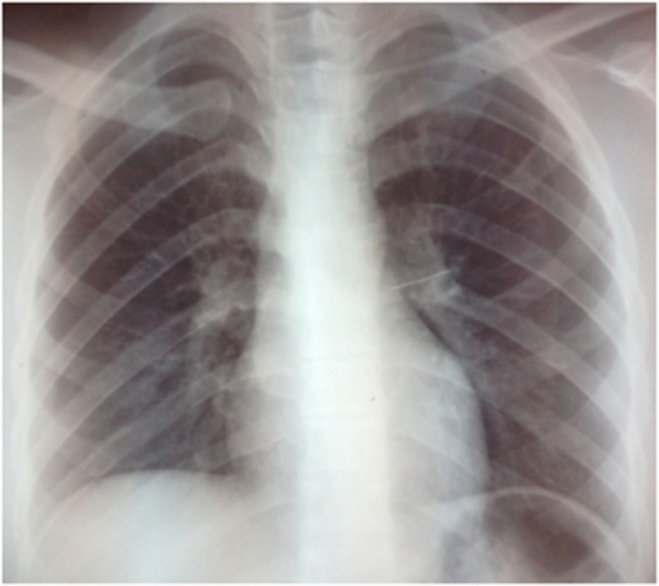
Chest radiograph showing a pin located in left superior lobar bronchus.

Chest radiograph was sufficient to determine the diagnosis, showing the pin in form of a linear opacity (Figure 1). It allowed us to localize pins that were found on the right side in 13 cases, and on the left in 15 cases. Inferior lobe was the most common location of pins (Table 1).

**Table 1:** **Distribution of pins in the tracheobronchial tree**

Right lung: 13 pins, being 43.5%	Left lung: 15 pins, being 56.5%
Superior lobe: 1	Culmen: 2
Middle lobe: 1	Lingula: 2
Inferior lobe: 10	Inferior lobe: 9
Main bronchus: 1	Main bronchus: 2

All patients were operated by posterolateral thoracotomy under general anesthesia with elective intubation. The intraoperative pin’s localization was based on careful palpation of lung parenchyma searching for the plastic ending. It is usually easy to find when the affected lung is collapsed. However, palpation alone was insufficient in 7 patients and additional intraoperative radioscopy was necessary in 5 cases and flexible bronchoscopy in 2 cases.

**Figure 2:**
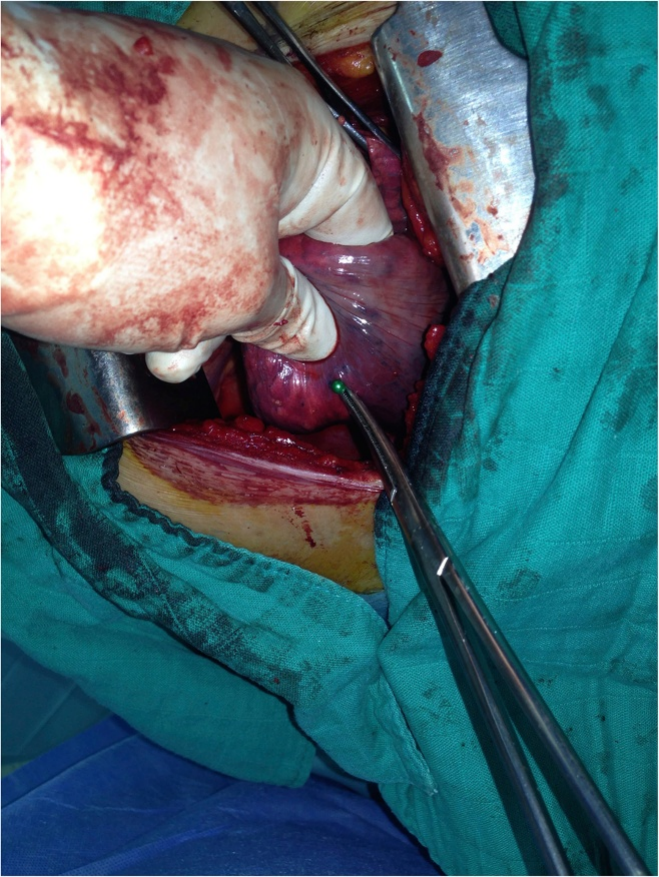
Removal of scarf pin by pneumotomy.

In all cases, surgery was conservative and no parenchymal resection was necessary. Removal of pins was performed by incision of the parenchyma or pneumotomy (Figure 2) in 16 patients who had peripheral FBs and by bronchotomy in 12 cases where FBs were central and/or embedded in the bronchus wall.

Post-operative recovery was uneventful in 90% of patients. Three patients have shown air leaking for more than 5 days. Average length of postoperative drainage was 3 days.

## Discussion

Foreign body (FB) inhalation is a serious accident that happens most commonly to children 3–4 years old and to adults above 50 years old, as they have tendency to swallowing disorders [1,5]. The nature of foreign body varies according to age, regions, eating habits and even clothing habits [1,2,4]. In our series all patients were young, 20 years old on average, which reflects data found in literature [1,2,4,5]. They were all wearing veil and inhalation was by accident while patients tried to place pins in their scarf and held one or more pins between their teeth [1,5]. Most patients reported that they were speaking, coughing, laughing or taking a deep breath at the time of accident. Their young age and lack of experience in arranging the veil played a favoring role as well [1,5].

Due to slender shape of pins, inhalation is never asphyxiating. Symptomatology can be summarized as a penetration syndrome (coughing fits, suffocation and dyspnea) [1,3,5]. In our series that syndrome was present in 82% of cases. These symptoms fade after a few minutes. Zaghba [5] and A. Hebbazi [1] experienced similar findings in their series. According to literature, the rate of asymptomatic patients is between 10% and 20% [1,6].

Complications of intrabronchial FB are obstructive emphysema, recurrent pneumopathy, bronchiectasis, pulmonary abscess, pleural effusion or even pneumomediastinum [7-9]. These complications are more common if delay between inhalation of FB and its removal is prolonged [5,8]. As pins are slender and metallic, they do not favor stasis, surinfections and parenchymal destructions. It explains the absence of all these complications in our patients. Otherwise, FBs embedded in the bronchial mucous membrane cause inflammatory reaction with granuloma formation around the FB which makes its endoscopic removal extremely difficult [1,5].

Chest radiograph is a simple and efficient test to determine diagnosis [1,5]. It was sufficient to diagnose our patients, as pins were shown as linear opacity.

Contrary to results of other authors [1,4,5], location of pins in left tracheobronchial tree was slightly predominant in our series; 15 cases (53.5%) as opposed to 46.5% on the right side. Frequency of left localization in our series can be explained by relatively easy endoscopic removal of pins located on the right. As a result, more patients with pins on left side needed surgical removal.

Flexible bronchoscopy is the best therapeutic means [10]. Preferably, it should be performed urgently before complications arise [11,12]. Success rate of this treatment varies according to authors: 56% for Al-Ali, 62.5% for Hebbazi and 80.6% for Zeghba [1,5,13]. In our series, average number of attempts of flexible bronchoscopy removal was 2.5 times per patient. It is difficult to estimate success rate of endoscopic removal as our patients came from different centers of Rabat region. In case of unsuccessful flexible bronchoscopy, rigid bronchoscopy may be very helpful [2]. It was used in case of 7 patients in our series. Reasons of unsuccessful endoscopy were: distal localization favored by characteristic shape of this FB, pins embedded in bronchus wall with inflammatory reaction.

In case of unsuccessful endoscopy, surgery is indicated. The rate of need for surgery in literature varies from 1.6 to 18% [6,7,14,15]. All our patients were operated by posterolateral thoracotomy. Choice of surgical procedure depends on the location of FB and the time since the inhalation. Pneumotomy, bronchotomy and parenchymal resections are the different surgical procedures [4,7]. In our series, treatment was always conservative, no resection was used.

In case of peripheral pins, perioperative localization by simple palpation of collapsed lung, was generally easy. In difficult cases, localization through endoscopy or radioscopy can be useful. These techniques were used in 7 of our patients.

## Conclusion

Scarf pin is a particular type of tracheobronchial foreign body. Preferred treatment is endoscopic removal. It has to be performed as soon as possible. Surgery is an alternative in case of unsuccessful endoscopy. It is associated with low morbidity. It has to be conservative as much as possible. Localization by careful palpation of collapsed lung parenchyma is the principal technique of this procedure. Perioperative radioscopy or bronchoscopy can be useful.

## Consent

Written informed consent was obtained from the patients for the publication of this report and any accompanying images.
